# Trade-Off between Toxicity and Signal Detection Orchestrated by Frequency- and Density-Dependent Genes

**DOI:** 10.1371/journal.pone.0019805

**Published:** 2011-05-19

**Authors:** Laury Arthaud, Selim Ben Rokia-Mille, Hussein Raad, Aviv Dombrovsky, Nicolas Prevost, Maria Capovilla, Alain Robichon

**Affiliations:** 1 UMR INRA/CNRS/UNSA 6243, University of Nice Sophia Antipolis, Sophia Antipolis, France; 2 Agricultural Research Organization, The Volcani Center, Bet Dagan, Israel; 3 Dulbecco Telethon Institute, Department of Biology and Evolution, University of Ferrara, Ferrara, Italy; Deutsches Krebsforschungszentrum, Germany

## Abstract

Behaviors in insects are partly highly efficient *Bayesian* processes that fulfill exploratory tasks ending with the colonization of new ecological niches. The *foraging* (*for*) gene in *Drosophila* encodes a cGMP-dependent protein kinase (PKG). It has been extensively described as a frequency-dependent gene and its transcripts are differentially expressed between individuals, reflecting the population density context. Some *for* transcripts, when expressed in a population at high density for many generations, concomitantly trigger strong dispersive behavior associated with foraging activity. Moreover, genotype-by-environment interaction (GEI) analysis has highlighted a dormant role of *for* in energetic metabolism in a food deprivation context. In our current report, we show that alleles of *for* encoding different cGMP-dependent kinase isoforms influence the oxidation of aldehyde groups of aromatic molecules emitted by plants *via Aldh-III* and a phosphorylatable adaptor. The enhanced efficiency of oxidation of aldehyde odorants into carboxyl groups by the action of *for* lessens their action and toxicity, which should facilitate exploration and guidance in a complex odor environment. Our present data provide evidence that optimal foraging performance requires the fast metabolism of volatile compounds emitted by plants to avoid neurosensory saturation and that the frequency-dependent genes that trigger dispersion influence these processes.

## Introduction

Environmental instability is known to induce the development of genetic variants in many taxa that have the capacity to enlarge their phenotypic plasticity range when confronted with challenging conditions. This may lead to the selection of stable genotype/phenotype profiles that are adapted to environments characterized by periodic changes [1]. In contrast, a frequency-dependent selection of alleles may lead to a transient prevalence of a trait depending on the actual state of the ecological niche [2], [3]. In such cases, the prevalent adaptive traits can be reversed in relation to environmental changes. Selection by both density- and frequency-dependent processes seems to be highly efficient in terms of adaptation as, even though a phenotype is prevalent, it is not fixed and might fluctuate depending on environmental changes [4], [5], [6].

**Figure 1:**
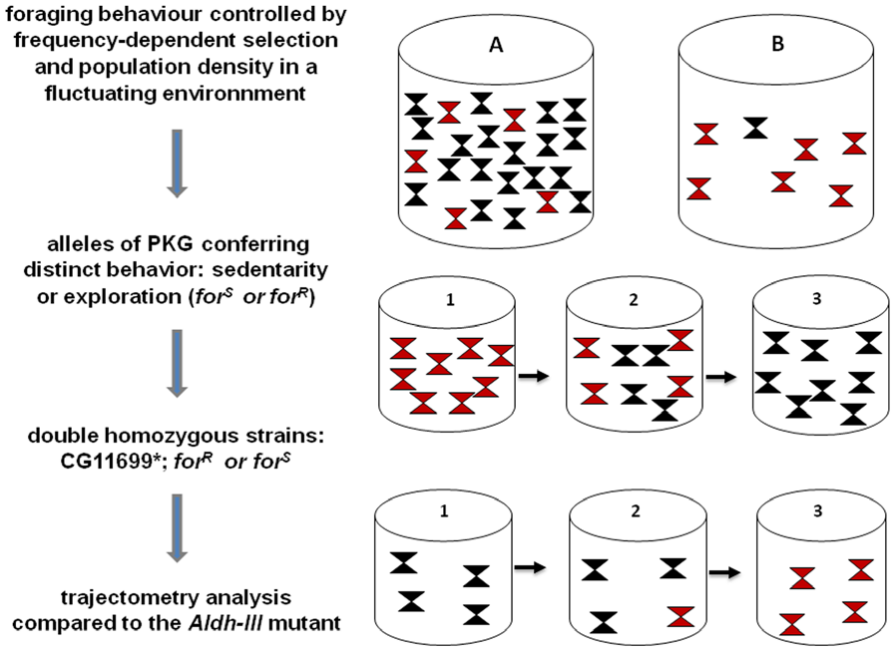
Experimental scheme used in this study and illustration of density-dependent selection of genes in relation to phenotype transmission. (*left*) Experimental design used to demonstrate the pleiotropic effects of the frequency- and density-dependent gene *for*. Double-hybrid system analysis has previously shown a strong probability of interaction (0.7) between CG11699 and Aldh-III and a lower probability (0.4) of association between CG11699 and PKG, the product of the gene *for* [15]. The genetic backgrounds (expressing specific sets of transcripts of *for*) conferring the bimodal behaviors Rover (exploratory) or Sitter (sedentary) were crossed with two *CG11699* mutants (containing P-element insertions) using chromosome balancers to construct the double homozygous strains CG11699*; *for^R^* and CG11699*; *for^S^* (see Table S1). These strains were then tested by trajectometry analysis using an arena connected to attractive odorants such as aldehyde compounds and compared with *Aldh-III* mutants. (*right*) A and B represent high- and low-density populations. Red and black triangles represent phenotypes determined by their corresponding expressed alleles (Rover, *for^R^*, black and Sitter, *for^S^*, red), dominant in each density context. The second line illustrates the flies from a low-density context raised at high density. The third line illustrates the opposite trend: flies from high-density context are raised at low-density. The progressive change of phenotypes is observed through few generations.

In our present analyses, we took advantage of a natural polymorphism that affects *Drosophila* foraging behavior and is controlled by high and low animal rearing densities. The exploratory (Rover) phenotype was selected for at high-density conditions, whereas the sedentary (Sitter) phenotype was selected for at low-density conditions [7], [8] (see figure 1 for illustration). These behavioral changes are induced by modifications of the expression of some isoforms of the *for* gene [9], [10] and this manipulation of polymorphic behavior by only one gene is caused by an assortment of cGMP dependent kinase isoforms, for which the mechanisms of switching remain unknown [9], [10]. Allelic variation has been reported to affect associative olfactory learning, with Rover showing a stronger short-term and a weaker long-term memory and Sitter manifesting the opposite trend (*i.e.*, a robust long-term and weaker short-term memory) [11]. Moreover, plasticity in metabolic responses, such as the modification of lipid synthesis, involves the *for* gene in the context of food deprivation and differs in the Rover and Sitter behavioral variants [12].

Because the *for* locus generates alternative behavioral phenotypes under environmental control, this gene model presents an opportunity to analyze genotype-by-environment interaction (GEI) effects, when the variants are placed under environmentally constrained conditions. In this scenario, microarray technology has revealed broad transcriptional differences between Rover and Sitter, specifically in a starvation context [12]. Again, the frequencies of the two modes of this behavioral binary trait will fluctuate over time and consequently PKG (*for*) isoforms maintained as a flexible hereditary trait are subject to reverse selection [7].

In a more spectacular mode, two distinct behavioral phases exist in other insect species, such as the solitary and the gregarious locust. The solitary phase characterizes sluggish individuals with a low metabolism that are camouflaged with the colors of the surroundings and morphologically have short wings and long legs [13], [14]. These insects transform into a gregarious state in crowded spaces with con-specifics, characterized by color changes, broad physiological modifications such as a highly active metabolism and drastic behavioral shifts. When the population is sufficiently dense, the compact migration phase commences [13], [14]. Unfortunately, no genetic or genome data have been reported with respect to this animal model. The characteristics of *Drosophila* are far removed from this amazing bimodal reversible shift in the locust. However, the Rover/Sitter dual behavior of *Drosophila*, which is mediated by the *for* gene, is reminiscent of the locust binary phases in terms of the alternative allele expression that is conditionally heritable and under the control of the environment *via* frequency- and density-dependent processes.

A previous seminal report has established the probability of *Drosophila* protein-protein associations based on high throughput analyses using the yeast double-hybrid system [15]. This study employed the full spectrum of expressed proteins and the results are provided with an attributed score that takes into account factors such as compartmentalization or whether the proteins are membranous or hydrophilic. This system revealed that the *for* protein binds to a small number of proteins, as the *CG11699* gene product with a medium score (0,4). CG11699 also shows a high probability of association with the *Aldh-III* protein (score 0,7), which suggests the existence of a trimeric complex, *i.e.*, For*/*CG11699*/*Aldh-III [15]. The predictive structure of CG11699 indicates that this small protein (15 kDa), with two transmembrane domains, has putative phosphorylation sites for PKG, PKA and PKC. Consistently, as *for* encodes a cGMP-dependent kinase, it is a candidate to control CG11699 through phosphorylation. CG11699 is currently of unknown function and presents sequence homology between orthologs in many species, including: insects (*Drosophila*, *Aedes egypti*, *Apis mellifera*, *Aphid acyrtosiphon* and *Anopheles gambiae*), a number of mammals (*Canis lupus familiaris*, *Mus musculus* and *Homo sapiens*) and birds (*Gallus gallus*).

Briefly, odorants from fruit in decomposition (on which fruit flies feed in nature) are mostly carboxylic compounds, alcohols and aldehydes [16]. More importantly, aldehydes are simultaneously toxic compounds and powerful attractive neuro-sensorial stimuli that guide flies in exploration and fix them on food resources [16], [17]. This is a dilemma for flies and other insects: to use toxic compounds as very specific ligands for olfactory signaling and to eliminate rapidly these compounds by biochemical processes. In fact, aldehydes are highly reactive groups promoting covalent bonds mainly with amine groups (lysine of proteins) through intermediate Schiff base [17], [18]. Moreover flies use alcohols like ethanol as a resource, but an excess of a conversion product (acetaldehyde, a metabolite which enters glycolysis for ATP synthesis) will cause damage. Finally, lipid peroxidation by oxygen attack on unsaturated lipids in the membrane leads to compounds like malondialdehyde (0 = CH- CH = CH-CH = 0) [16], [17]. Malondialdehyde is highly toxic because it bridges proteins as glutaraldehyde does and is inactivated by *Aldh-III*, called also the fatty acid aldehyde dehydrogenase or the membrane cleaning enzyme [19]. As a consequence, flies, like many insects species, have to live in a permanent paradox: some molecules are powerful neurosensory stimuli to guide exploration and food searching, but on the other side they are also highly toxic if the detoxification process turns out to be inefficient.

In our current study, we generated *Drosophila* Rover and Sitter genetic backgrounds carrying homozygous *CG11699* mutations by P-element insertions. We designed behavioral tests using a trajectometry methodology to address the influence of *for* on the benzaldehyde responses by comparing the attractiveness and repulsion scores of the double homozygous *Drosophila* mutants CG11699*; *for^R^* and CG11699*; *for^S^* with those of *Aldh-III* hemizygous mutants. *Aldh-III* is an aldehyde dehydrogenase family member with nine transcripts in *Drosophila* and mutants are homozygous lethal. The interactions between the *for* and *Aldh-III* products through the phosphorylation of CG11699 were also investigated by transfection of *Drosophila* cultured cells with an expression vector bearing the coding sequence of a tagged *CG11699* product. Benzaldehyde at a low concentration is an attractant, whereas at high concentrations it is a strong repellent [20], [21], [22], [23]. The present data suggest strongly that dispersion induced by density-dependent genes simultaneously reinforces the metabolism of xenobiotics that guide exploration.

## Results

### Loss of function in exploratory tests suggests a CG11699/Aldh-III interaction

**Figure 2:**
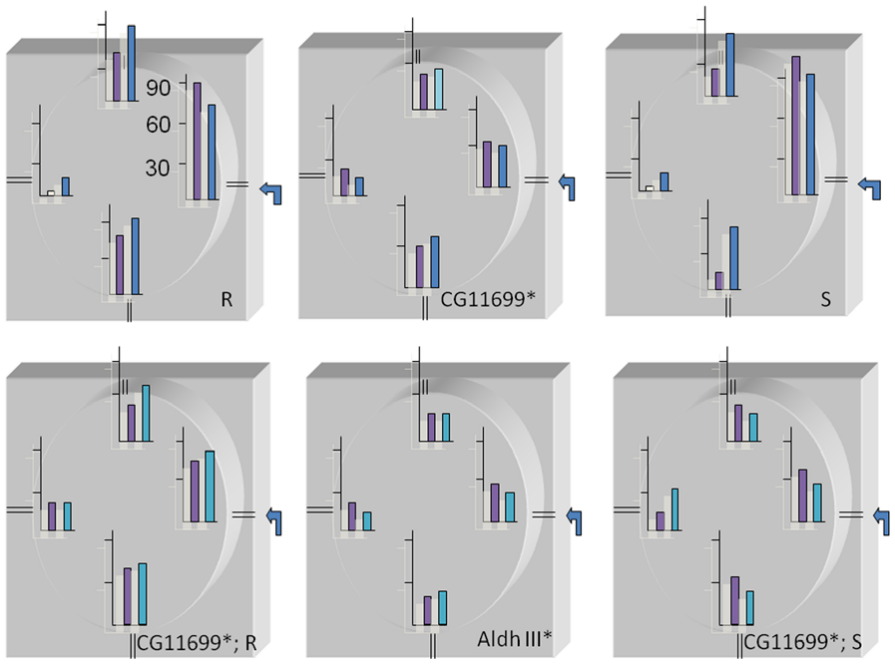
Benzaldehyde-induced response of double homozygous mutants bearing a *CG11699* allele in a Rover or Sitter genetic background conferring distinct dispersive behavior. Ten flies (five days old females in purple, males in blue) were starved for two hours before the start of the experiments and were then tested one by one. For quantification, four landmarks as described in Figure S1 were used to count the number of passages. The cumulative frequency of passages is shown. These experiments were of five minutes in duration, during which time the flies are walking (the periods of time during which the flies were immobile were not counted). R indicates the Rover background and S denotes the Sitter background (two variants of the *for* gene). CG11699* indicates a strain with a P-element insertion in this gene. CG11699*; *R* and CG11699*; *S* represent double homozygous strains (P-element insertions in *CG11699* in the Rover or Sitter background). The scale of the ordinate axis (Y) represents 30, 60 and 90 passages. The protocol is described in Materials and Methods and in Figure S1. A mutant of *Aldh-III* (CG11140*) is shown for comparison. Mainly, Rover and Sitter gave similar results compared to *Canton S* (not shown): 90 and 85 passages for *Canton S* females and males respectively at the check point corresponding to the arrival of odorants *versus* 90 and 84 for R, 100 and 92 for S. These numbers of passages fall drastically for Aldh-III* (25 and 20), CG11699*(35 and 30), CG11699*; *for^R^* (45 and 50) and CG11699*; *for^S^* (35 and 25). Statistical analysis was performed using Paired *t*-test on individual performance of ten flies for each strain and results are reported in table S2.

**Figure 3 pone-0019805-g003:**
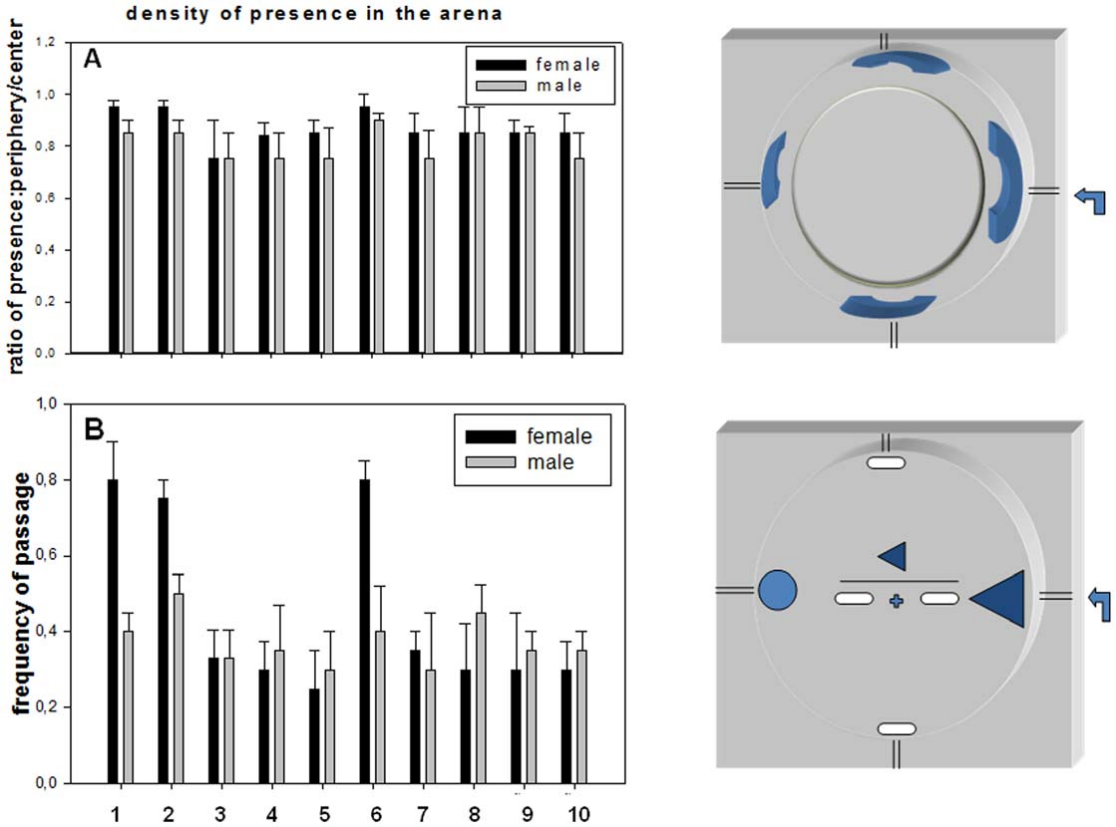
Comparative propionaldehyde-induced behavioral response between strains. Ten five days old female (black) and male (grey) flies were tested after two hours of starvation for propionaldehyde-induced exploration using the arena/air pusher system. This series of experiments was conducted during the odor gradient phase generated in the arena prior to reaching a uniform concentration (equal concentration between syringe and arena). Flies were tested for ten minutes, during which time they were active. (A) The ratio of time spent in the periphery *versus* the inside circle is indicated in the graph for the strains tested (1 corresponds to the entire time spent in the arena). (B) In another series of experiments, the passages in front of the two white oblong shapes and of the triangle were counted. The graph represents the ratio of passages in the triangle to the total (1 =  triangle *plus* the two white oblong shapes). Bars represent the mean +/− S.E. for ten flies (females or males). 1, *for^R^*; 2, *for^S^*; 3, Aldh-III*; *4*, CG11699* (Szeged stock P-element insertion in *CG11699*); 5, CG11699**, [*(EP)EP* Berkeley project, P-element insertion in *CG11699*)]; 6, *CS*, *Canton S*; 7, CG11699**; *for^S^*; 8, CG11699**; *for^R^*; 9, CG11699*; *for^S^* and 10, CG11699*; *for^R^*. Mainly, the ratio of passages in the odor entry landmark (triangle) gave, respectively for females and males: *for^R^*, 0.8+/−0.1 and 0.4+/−0.05; *for^S^*, 0.75+/−0.05 and 0.5+/−0.05; Aldh-III*, 0.35+/−0.05 for both females and males; CG11699*, 0.3+/−0.05 and 0.38+/−0.1; CG11699*; *for^R^*, 0.3+/−0.15 and 0.35+/−0.1; CG11699*; *for^S^*, 0.3+/−0.1 and 0.35+/−0.1. Statistical analysis was performed with a Paired *t*-test on ten individuals for each strain and is reported in table S2.

**Figure 4:**
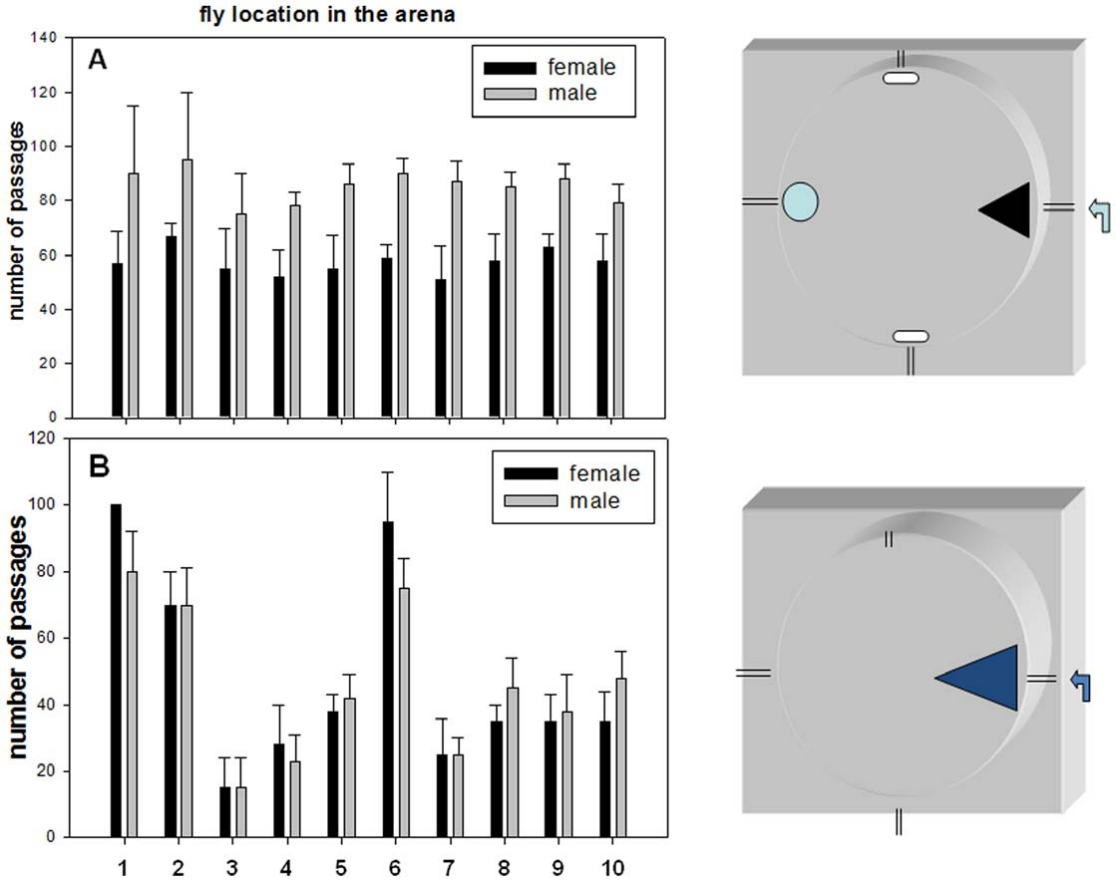
Comparison of the exploration characteristics between strains stimulated by a cocktail of aldehyde compounds. Five day old female and male flies were tested after two hours of starvation for their behavioral response using the arena/air pusher system and a cocktail of benzaldehyde/propionaldehyde/acetaldehyde at low concentrations (see Materials and Methods). The Y axis refers to the number of passages of individual flies in the four arena landmarks as indicated in the drawings. The experiment and the measurements were conducted in conditions where the flow of aldehyde compounds/air from the syringe creates a gradient in the arena. (A) For one series of experiments, the cumulative passages across the four landmarks were counted for a discontinuous period of ten minutes during which time the flies were active (discontinuous periods of time in which flies were immobile were not counted). Numbers represent the addition of three tested flies and bars represent the mean of five repeat experiments +/− S.E. (each repeat is the addition of three tested individual flies). (B) For another series of experiments, the passages in the blue triangle corresponding to the odor entry were counted. The discontinuous timing during which female Rover strain flies were active was measured for 100 passages. The average of ten Rover females was a time reference during which the other strains (female and male) were comparatively tested. The bars represent the mean+/− S.E. of five individual flies. 1, Rover; 2 Sitter; 3, Aldh-III*; 4, CG11699*; 5, CG11699**; 6, *CS*, *Canton S*; 7, CG11699*; *for^S^*; 8, CG11699*; *for^R^*; *9*, CG11699**; *for^S^*; 10, CG11699**; *for^R^*. Mainly, the number of passages at the odor entry landmark gave: Rover, 100 (reference) and 80+/−15 for female and male respectively; *CS*, 95+/−18 and 80+/−10; *S*, 70+/−10 (female and male); CG11699*, 25+/−15 and 20+/−10; CG11699*; *for^R^*, 30+/−5 and 45+/−10; CG11699*; *for^S^*, 25+/−10 and 25+/−5. The statistical analysis using the Paired *t*-test was performed on the tested groups and is reported in table S2.

The rationale behind the experiments carried out in this study is to establish a link between olfaction-based exploration and an interfering molecular pathway involving the *for* and *Aldh-III* genes. Both *for* and *Aldh-III* present multiple transcripts (see Figure S1). The *CG11699* gene product was shown to interact with For and Aldh-III in the double-hybrid system [15]. We constructed double homozygous mutants with the *CG11699* gene modified by a P-element insertion in both the Rover and Sitter genetic backgrounds, which differ in encoding different sets of *for* transcripts (see Figure S1). The experimental approach and the exploratory protocols used to test these strains are depicted in Figure 1 and in Figure S1. Briefly, odors are introduced into an arena through a capillary tube fixed on one hole (arrow) using a syringe pusher (constant flow). Four holes (to avoid overpressure), of a size slightly lower than that of a fly body, are positioned at equal distance (double traits in figures 2 to [Fig pone-0019805-g003] 4). A landmark was attributed to each of the holes and passages of flies were counted. Trajectometry analysis was performed to measure the intensity of searching and/or exploring reported as the number of passages in landmarks when flies are exposed to an attractive concentration of aldehyde coming from a hole. The number of passages nearby the odorant entry was analyzed as the fly effort to reach the source. Similar parameters measured between strains, like the restrictive spatial occupation in the arena or the global number of passages in the four landmarks, were used as internal controls of the procedure. Slightly different protocols were used in three different sets of experiments reported in figures 2, 3 and 4 in order to establish the validity of the observed phenotypic trends. The cumulative number of passages of the male and female flies, tested individually, was analyzed for each of the *for^R^*, *for^S^*, CG11699* strains and relative double homozygous genotypes in addition to *Aldh-III* mutants. We used benzaldehyde as aldehyde source due to the fact that it constitutes the best substrate for Aldh-III [24] and that it induces a bimodal response in *Drosophila* flies depending on the dose, *i.e*., at low concentration it is attractive and at high concentration it is repulsive [21]. The numbers obtained for female and male *Canton S* (*CS*) flies, shown in Figure S1, constitute a reference for the series of reported experiments. In summary, the *Aldh-III* mutants exhibit a drastic decrease in exploration in accordance with a strongly altered benzaldehyde metabolism (Figure 2). The *CG11699* mutant flies are affected at levels in between those of Rover and Sitter and those of *Aldh-III* mutants (Figure 2). We observed also that the profiles of the Rover and Sitter flies are significantly modified by the homozygous introduction of the *CG11699* mutation (Figure 2). It seems that the double mutants have the same phenotype as the *CG11699* single mutants, thus suggesting that the *CG11699* mutation is not by-passed by *for*. This trend was also confirmed using another *CG11699* mutant (see Supplementary Data, figure S2). The *for^R^* and *for^S^* flies were not distinguishable in their foraging pattern when we compared the number of passages in front of the odor entry. This test does not appear sensitive enough to highlight these two natural phenotypic differences. However, the density of passages in front of the four holes is dissymmetric for *for^R^*, *for^S^* and *CS* whereas it tends to be uniformly distributed for the *Aldh-III* and *CG11699* mutants. These drastic differences were also observed with the double homozygous mutants, which show unambiguously a profile resembling that of the *Aldh-III* mutant.

Moreover, the trajectometry profiles were analyzed using another aldehyde-based compound, propionaldehyde, which results in the same bimodal response as benzaldehyde [20], [21]. This compound essentially produced behavioral results that were similar to those obtained with benzaldehyde (Figure 3).

Sexual behavioral dimorphism was observed for *Canton* S, Rover and Sitter controls with the two aldehyde compounds, and this seemed to be abolished in the *CG11699* mutants and double homozygous mutants (Figures 2 and 3). Moreover, through these experimental sets, Rover males showed a different profile than Sitter males: the former seemed more aggressive in searching and partially unfocused on the odor entry (Figure 3). Finally, a further experiment was conducted with a cocktail of three aldehyde compounds at low concentration, *i.e.*, benzaldehyde, propionaldehyde and acetaldehyde (Figure 4). Again, the trajectometry trend accorded with the preceding experiment, suggesting strongly that the *Aldh-III/CG11699* pathway intervenes in aldehyde recognition, odorant-stimulated exploration and spatial guidance.

**Figure 5:**
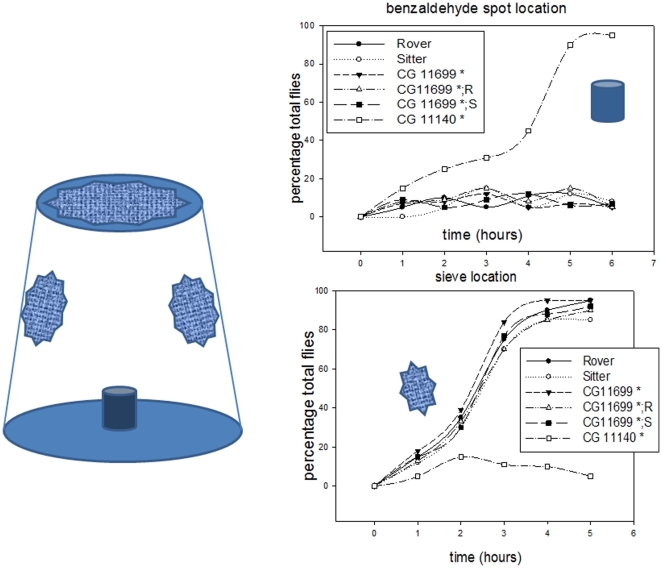
Behavioral responses induced by a high concentration of benzaldehyde. One hundred flies (female and male, five days old) were transferred to a cage as shown on the left. This cage was then placed in a hood to enable a constant fresh air flow in contact with the sieves. A 50 µl aliquot of benzaldehyde was deposited on a plug made of foam rubber inside the cage. This high concentration induces deep sleep in *Drosophila*. The graphs represent the percentages of flies that are asleep, aggregated or immobile either on the plug (top) or on the sieves (bottom) during the time course of the experiment. Rover, *for^R^*; Sitter, *for^S^*; CG11699*, P-element insertion in *CG11699* (Bloomington Stock Center); CG11140*, *Aldh-III* mutant. CG11699*; *R* and CG11699*; *S* are the double homozygous mutants. Plots are the average of five experiments. CG11140* gave about 100% of flies aggregated on the plug and 0% on the windows at the equilibrium. The numbers were opposite for the other strains (about 0% and 100% respectively).

Experiments using strong concentrations of benzaldehyde were also conducted because this induces a deep sleep after a variable period of exposure (the flies are immobile on their legs, differently from CO_2_-induced anesthesia). The time course of the accumulation of immobile flies at different places in the device is shown in figure 5. Most of the strains were found to localize on the windows of the cage at the benzaldehyde/fresh air interface whereas the *Aldh-III* mutants are preferentially located on the benzaldehyde plug. Amazingly, the *Aldh-III* mutants showed a strong attractiveness for the high concentration of benzaldehyde, which was not observed with the other strains examined in this study (Figure 5). The mechanisms underlying this phenomenon remain unknown, but our observations highlight the fact that enzymatic degradation of aldehyde compounds participates strongly in the neurosensory signaling pathways that drive attractiveness/repulsion coupling.

To address whether complex natural flavors might reproduce the same type of behaviors as those described above, trajectometry analysis was conducted using grape juice odorants. The results showed very few differences among the strains, which indicates that the behavior of the mutants is specific to aldehyde compounds and does not involve all aromatic molecules that might exist in the natural ecological niches of *Drosophila* (see Supplementary Data, figure S3). Overall, these data suggest that the alleles of *for*, which encode cGMP kinase isoforms responsible for the dispersion phenotype, will not overcome the deficiency of *CG11699* in aldehyde sensory behavioral tests.

### Evidence for a role of phosphorylated CG11699 in membrane aldehyde dehydrogenase activity

**Figure 6:**
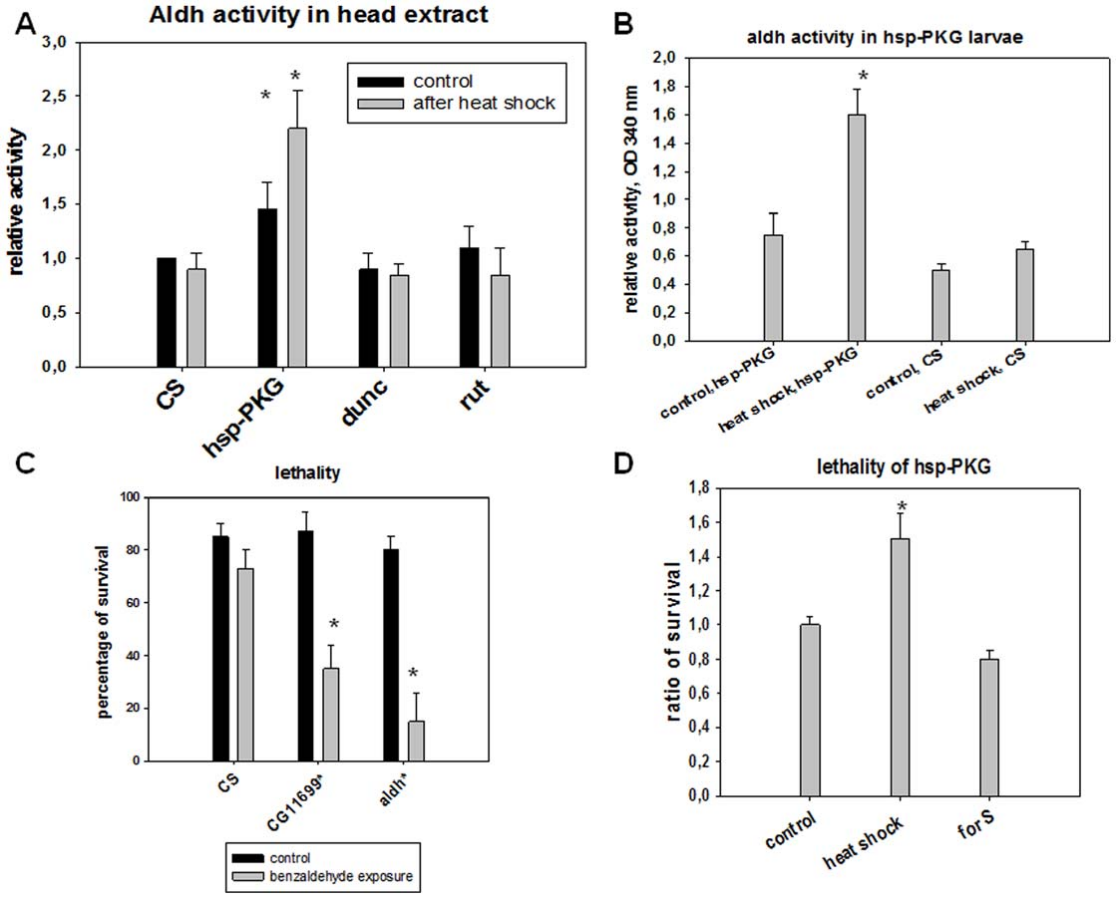
Aldh activity analysis in strains presenting high or low levels of PKA or high levels of PKG and effect of benzaldehyde exposure on the lifespan of flies. (A) Ten flies (female and male), submitted (grey) or not (black) to heat shock, were decapitated and the membrane head extracts were assayed for Aldh activity using 100 µg of membrane proteins and benzaldehyde as substrate (see Materials and Methods). The *dnc* mutant has high levels of cAMP due to a defect in phosphodiesterase. The *rut* mutant presents a low level of cAMP due to a cyclase defect. *hsp-PKG* is a transgenic fly bearing the *for* transgene responsible for the dispersion behavior under the heat shock promoter in a Sitter background. The determination obtained with the *CS* extract was a reference to compare the other strains. Bars are the mean of five experiments +/−S.E. *p<0,001. (B) Aldh activity in third instar larvae in the *hsp-PKG* strain with or without heat shock prior to the dosage. Bars represent the mean of 3 experiments+/−S.E., *p<0,001. (C) For the lethality, 50 five days old flies (female and male) were exposed to a high concentration of benzaldehyde in a food vial for five minutes (10 µl benzaldehyde deposited on paper) and this was repeated three times on day 5. Lethality was counted one week after. Aldh-III* and CG11699* mutants were assayed versus *CS* as control. Bars are the mean of five experiments +/−S.E., *p<0.0005 Student's *t*-test for benzaldehyde exposure of Aldh-III* and CG11699* versus CS for the heat shock experiments. (D) Third instar *hsp-PKG* larvae (50 larvae) with or without heat shock were exposed to a high concentration of benzaldehyde (10 µl benzaldehyde deposited on paper, 3 times) in a food vial for one day. Then, the flies that emerged from pupae were counted. Bars are the mean of three separate experiments +/−S.E., *p<0,005 and represent the ratio of surviving adult flies *versus* the control (*hsp-PKG* without heat shock). The *for^S^* strain is shown as the background in which *hsp-PKG* transgene has been introduced.

Following the published double-hybrid data that provide evidence of an interaction between the *for* gene products (encoding cGMP dependent kinases) and the *CG11699* protein on one side, and the *CG11699* protein with *Aldh-III* proteins on the other side, we decided to analyze Aldh activity and fly survival in transgenic flies presenting inducible amounts of kinase (PKG) corresponding to the isoform of *for* responsible for the dispersion behavior [9], [25] under the control of cGMP (*hsp-PKG* transgenic flies). As a control, high and low levels of PKA activity, respectively in *dunce* (*dunc*) and *rutabaga* (*rut*) mutants (*dunc* has an invalid phosphodiesterase responsible for a high level of cAMP and *rut* has an invalid cyclase characterized by low levels of cAMP [26]), were tested for their Aldh activity in parallel with *hsp-PKG* flies. Results are summarized in figure 6. The level of PKA activity seems to have no effect on the level of Aldh activity (Figure 6A); in contrast, the over-expression of PKG increases significantly the amount of Aldh activity in adults (Figure 6A) and in larvae (Figure 6B). Moreover, the lethality analysis of the *CG11699* and *Aldh-III* mutants after acute exposure to benzaldehyde demonstrates the role of detoxification processes to maintain fly lifespan that involve aldehyde dehydrogenases (Figure 6C). The overexpression of PKG (Rover allele) in larvae prior to acute benzaldehyde exposure increases the survival of emerged adults from pupae, which confirms the role of this kinase in aldehyde detoxification processes (Figure 6D).

**Figure 7:**
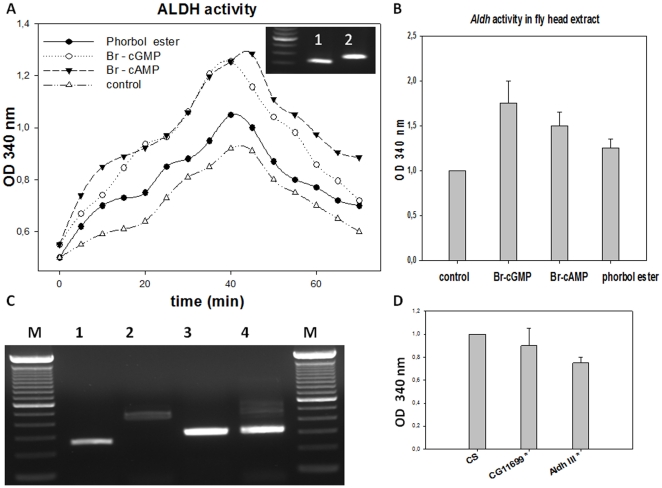
Aldh activity analysis of S2 cells after treatment with kinase activators. (A) Aldh activity using benzaldehyde as substrate was determined by measuring the spectrophotometric absorbance (OD) at 340 nm, which specifically quantifies the amount of reduced co-enzyme (NADH, NADPH). The main graph is representative of the Aldh activity from cells previously treated with 50 µM Br-cGMP, 50 µM Br-cAMP or 1 µM phorbol ester. These stable pharmacological agents activate the PKG (encoded by *for*), PKA and PKC kinases, respectively. The curves are the means of three separate determinations. Inserted above the curves, is the PCR analysis of the transcript of *CG11699* (1) in the sub-clone of S2 cells used in this series of experiments and of its genomic fragment (2) (see figure S4 for the primers). To summarize, Br-cGMP treatment gave 1.3 *versus* 0.9 for the control. (B) The graph represents the comparative mean+/− S.E. of five determinations of the total activity in membranes of the adult *CS* heads. The pharmacological agents *plus* ATP (50 µM) and Mg^++^ (1 mM) had been previously incubated with the crude extract prior to the enzymatic determinations. The *Drosophila* head membrane extracts gave 1.8+/−0.25 for Br-cGMP treatment *versus* 1.5+/−0.15 for Br-cAMP and 1.2+/−0.1 for phorbol ester. Control *versus* Br-cGMP Student's *t*-test: p<0.005. (C) PCR analysis using total RNA from S2 cells used in this series of experiment was performed to determine the presence of *Aldh-III* transcripts. 100 bp markers (Invitrogen) were used (the most intense band is at 600 bp). Lane 1, *Aldh1*/*Aldh5* corresponding to transcripts A–H (240 bp); lane 2, *Aldh2*/*Aldh5* corresponding to transcripts C, G and H (430 bp); lane 3, *Aldh3*/*Aldh6* corresponding to transcripts A and C (300 bp); lane 4, *Aldh4/Aldh6* corresponding to transcripts A, C, and H (300 bp). See figure S4 for the design and the location of the primers. (D) The comparative dosage of Aldh activity was also determined with 100 µg of total membrane proteins from extracts of *CS* (used as control), CG11699* and Aldh-III* mutant adult flies (five days old) using the same protocol. Bars represent the mean of three separate experiments +/− S.E.

To address the question of the probable interaction between For and CG11699 acting on *Aldh-III* proteins, we designed biochemical experiments as follows. The S2 *Drosophila* cells naturally express most of the *Aldh-III* gene transcripts and, in some sub-clones, *CG11699* transcripts have been detected as well. We used the opportunity offered by a sub-clone that expresses endogenously both *Aldh-III* (Figure 7C) and *CG11699* (Figure 7A, top right) transcripts to address whether second messengers might have a regulatory role on Aldh-III activity. The prediction analysis of the putative phosphorylation sites in CG11699 gives significant scores for PKG, PKA and PKC (see Supplementary Data, figure S4). For these reasons, cells were treated with pharmacological agents that activate durably these major kinases to address whether Aldh-III activity is modulated by phosphorylation. PKG, PKA and PKC are ubiquitous kinases present in any type of cell and the drug treatments essentially erase the substrate-specificity of the isoforms due to overlapping effects of long-lasting activation. Substantial increase in Aldh activity was observed with Br-cGMP and Br-cAMP (for which the respective specificity for PKG and PKA overlap), and to a lesser extent with phorbol ester (a PKC activator) (Figure 7A). These data were corroborated by the direct measures of the activity in head extracts treated with the same pharmacological agents, which results were in accordance with those obtained with S2 cell cultures (Figure 7B). On the other hand, the direct measure on head extracts of the *Aldh-III* mutants shows a modest decrease of activity (figure 7D): this can be easily explained by the fact that flies have four major families of aldehyde dehydrogenase proteins and that the *Aldh-III* recessive lethal mutant still has a copy of Rover chromosome.

**Figure 8:**
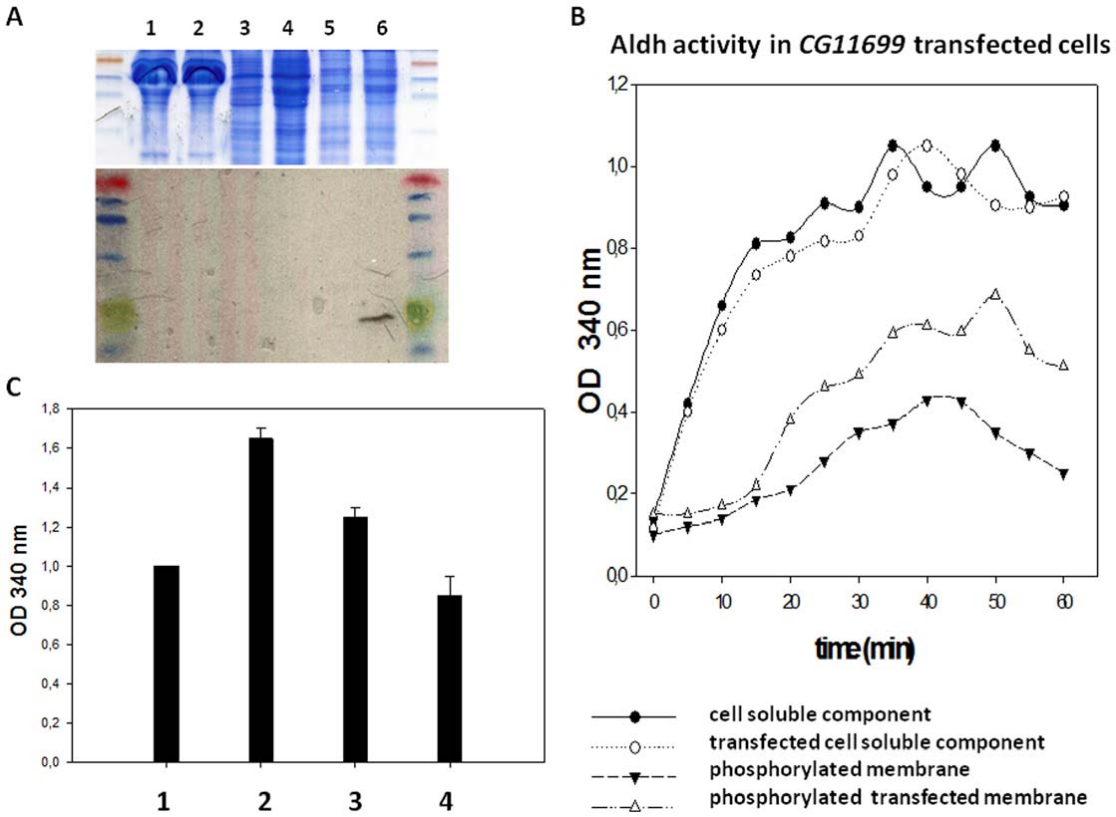
Aldh activity analysis of S2 cells stably transfected with a *CG11699* transgene. S2 cells were stably transfected with an expression vector bearing the *CG11699* coding sequence. A stable cell line expressing *CG11699* was generated by transfection with a plasmid containing CG11699-pMT/V5-His and pCoHygro (the *E. coli* hygromycin-B phosphotransferase gene under the control of a *Drosophila* Copia promoter). Stable cells were treated with 0.5 mM CuSO_4_ for 24 hr to induce *CG11699* expression before use. (A) Electrophoresis protein gel analysis: lane 1, total extracts of control cells; lane 2, total extracts of transfected cells; lane 3, soluble extracts of transfected cells; lane 4, soluble extracts of transfected cells after induction; lane 5, membrane extracts of transfected cells; lane 6, membrane extracts of transfected cells after induction. A Western blot analysis shows the presence of the expressed transgene using anti-V5 tag in the membrane fraction. (B) The induction of stably transfected cells was carried out one day prior to Aldh activity analysis. The cells were then disrupted by osmotic shock followed by brief sonication. The resulting extracts were briefly centrifuged to remove nuclei and organelles. The supernatant was again centrifuged to pellet the membrane fraction (13000 rpm for 30 minutes at 4°C) and the Aldh dosage was carried out for both components (soluble and membrane). The same amount of protein was used for the soluble fraction (100 µg) and for the membrane fraction (200 µg). Membranes were incubated or not with PKA subunit (10 units), ATP (50 µM) and Mg^++^ (1 mM) prior to Aldh activity determination. The curves represent the average of three determinations. (C) Comparative levels of Aldh-III activity in the membrane component from stably induced transfected cells, incubated or not with Br-cGMP and/or Br-cAMP three hours before the experiment. 1, control induced transfected cells; 2, induced transfected cells treated with Br-cGMP; 3, induced transfected cells treated with Br-cAMP; 4, control non transfected cells (with the inducer) treated with Br-cGMP. The bars represents the mean+/− S.E. of three individual determinations. p<0.001 Student's *t*-test between tracks 1 and 2. We observed elevated levels of Aldh activity after Br-cGMP treatment (0,8 +/− 0,15 for the non transfected cell control treated with the inducer *versus* 1,7+/− 0,15 for the induced transfected cells after br-cGMP treatment in both cases).

To obtain further insights into the underlying mechanisms, we attempted some biochemical analyses with purified proteins. *CG11699* was cloned into an expression vector for the stable transfection of S2 *Drosophila* cells. This allowed us to obtain a tagged protein containing histidine and V5 segments (Figure 8). The purification of the transgene product from the *CG11699* transfected cells proved to be extremely difficult to achieve and pull-down experiments of the active complex For/CG11699/Aldh-III failed. CG11699 and Aldh-III are membrane proteins and their solubilization in non-ionic detergent may have denatured their 3-D structure. Alternatively, we used transfected cells expressing a tagged *CG11699* protein to address the role of phosphorylated CG11699 upon endogenous Aldh activity. We found that phosphorylation of the membrane proteins of stably transfected cells with a high concentration of PKA subunit (at high level this kinase has overlapping activity with PKG) clearly increased the endogenous aldehyde dehydrogenase activity after induction of the *CG11699* transgene (Figure 8B). The amount of Aldh activity in the membrane was also more elevated when the induced transfected cells bearing the *CG11699* transgene were incubated with drugs as Br-cGMP, compared to control cells treated with the same drugs (Figure 8C). Moreover the co-application of PKA and a peptide inhibitor of this enzyme or an inhibitor specific to PKC clearly prove the role of phosphorylation accounting for the increase of Aldh activity (see figure S4). Although biochemistry with pure proteins was not achieved, indirect evidence using CG11699 enriched membranes supports the hypothesis that phosphorylation of CG11699 by PKG regulates Aldh-III activity.

## Discussion

In terms of the phenotypic plasticity observed in many insect species, the locust exhibits the most spectacular behavioral shifts, depending on social interactions in a high population density context, leading to an orchestrated migration to new ecological niches and feeding areas [27], [28], [29], [30]. Crowding appears to be a main cause of their drastic behavioral transformations *via* emitted chemicals that influence the development of juveniles [27], [28], [29], [30]. In *Drosophila*, similar bimodal behaviors, referred to as Rover and Sitter, are controlled also by population density [7]. The genetic tools available in *Drosophila* allowed us to examine the role of an over-expressed Rover transcript of *for* in relation with xenobiotic metabolism. Our experimental design also enabled us to compare the behavior of double-homozygous mutants bearing the Rover or Sitter variants and the *CG11699* gene disrupted by a P-element insertion. We show that these genotypes reproduce, at least to some extent, the olfactory behavior of the *Aldh-III* mutants. Biochemical analysis allowed us to conclude that Aldh-III activity is increased by phosphorylation *via* PKG (the gene product of *for*) and to a lesser extent PKA and PKC. This was corroborated by the multiple putative phosphorylation sites found in the cytosolic domain of the *CG11699* protein, based on the consensus sequences for the major kinases [31]. Because the *Drosophila* mutants used in this study were raised in a common environment during our present experiments, the genetically-based losses of function we observed argue for an interactive link between *for*, *CG11699* and *Aldh-III* (see Supplementary Data figure S5 for a proposed model). The differential tissue-specific expression and substrate-specificity of the isoforms of *Aldh-III* are little known. The impact of this gene on *Drosophila* development is equally poorly documented. Similarly, the kinase proteins that are generated alternatively by the gene *for* (Rover *versus* Sitter) are expected to exhibit major differences in their respective collection of phosphorylable targets. This differential “landscape” of phosphorylation is not known, due to technical and methodological limitations. Therefore, at this stage, it is difficult to go further to investigate the dialogue between the isoforms of *for* and *Aldh-III*. The powerful genetic tools available in the *Drosophila* model allowed us to deconstruct indirectly this scenario through the use of mutants and behavioral testing, thus bypassing extensive and extremely difficult biochemical studies. Finally, the *Aldh-III* gene is recessive lethal (viable in heterozygosity) despite the multiple aldehyde dehydrogenase isoforms issued from four genes. Considering that their enzymatic specificities overlap, the strong lethality observed with this mutant submitted to acute doses of benzaldehyde suggests a specific (if not exclusive) *Aldh-III* expression in crucial neuronal cells involved in exploration, odor guidance and survival in a toxic environment.

In mammals, the expression of *Aldh-III* homologues is either constitutive or inducible [32], [33], [34]. Mutations in the *Aldh-III* gene in humans that have been characterized over the last decade are responsible for the rare heritable neurocutaneous Sjögren-Larsson syndrome [19] (causing mental retardation and scaly skin). It is thought that the *Aldh-III* enzyme neutralizes the membrane aldehydes before they have a chance to form Schiff bases with amines of the membrane proteins. *Aldh-III* induction is triggered by the neoplastic transformation of the liver in mammals and also after exposure to the highly carcinogenic compound 2,3,7,8-tetrachlorodibenzo-p-dioxin (TCDD) [35], [36], [37]. The 5′ region of this gene contains several putative transcription factor binding elements, including a xenobiotic response element (XRE), an Ap1 binding sites, and a single Sp1 site [35], [36], [37]. A large panel of polycyclic aromatic compounds activate the vertebrate *Aldh-III* gene *via* direct binding to a transcription factor in conjunction with a protein partner (ARNT) [36]. Aldh-III represents one third of the total protein in the cornea in mammals [38]. This abundant enzyme eliminates the aldehyde groups generated by UV light, thus preventing covalent binding with the amino groups of proteins and the hazardous consequences for vision that this would otherwise entail. This emphasizes the sophisticated regulation of a gene by the coincidental integration of multiple modifiers. It also highlights how a gene under frequency- and density-dependent regulation influences the aldehyde metabolism to balance toxicity and neurosensory signaling.

Thus, frequency-dependent selection has evolved in unpredictable and disruptive environments and this may lead to discrete polymorphisms operating as an adaptation mechanism. The fitness of a trait can change over time depending on the population density, the abundance/rarefaction of resources and/or other environmental factors. Rare variants may have a high fitness in a novel environment and as a result will become dominant over time, whilst the normally dominant variant in a precedent phase will become rare. In parallel to our studies, frequency-dependent selection has been mostly described for genotypes in the context of pathogen/host relationships [39]. As another example of the diversity of these phenomena, social bees, wasps, and ants can modify their sex ratios in order to optimize the fitness of the colony [40], [41]. Diploid individuals that are heterozygous at the sex locus develop into females, whereas haploid and/or homozygous diploid individuals develop into males [42]. The underlying mechanisms behind these different examples illustrating the same paradigm are largely unknown. They are adaptive and reflect the ecological constraints at the time.

On the other hand, an abundant literature describes phenotypic changes when new conditions emerge raising alternative scenarios to Darwinian genetics, as highlighted by flourishing new reports on GEI [43], [44], [45], [46], [47], [48], [49]. Some acquired phenotypes might come from heritable but unstable traits, which could be relevant to a system of epigenetic heritable transmission (Figure S1) [50], [51]. The transcript variations associated with the *for* gene might come from a transposon inserted in the first intron, depending on its epigenetic methylation state controlled by the environment [52]. Consistently, no differences in gene sequence were detected between Rover and Sitter flies, which suggests that the genotype by itself can not explain the observed polymorphism. Alternative epialleles that are conditionally heritable might lead to this adaptive behavior.

Another hypothesis is that the *for* gene might influence aldehyde metabolism in other physiological contexts. For instance, flies recover from complete anoxia after four hours in contrast to humans, in whom death occurs quickly in the absence of oxygen [53], [54], [55]. This highlights the insect ability to activate alternative ATP synthesis pathways beside mitochondrial oxidative phosphorylation. Regardless of the activated metabolic routes, the anaerobic energy production in flies likely requires alternative modes of acetate/acetyl CoA production via the conversion of acetaldehyde by the *Aldh* family [53], [54], [55].

In this report, we have shown that the dispersive phenotypes are better equipped to metabolize aromatic compounds emitted by plants, thereby making the exploration process more efficient. Flies appear to have developed a “trade-off” system to balance the physiological role of xenobiotic compounds and their toxicity. Fly exploration supposes strong detoxification of deleterious molecules, which are paradoxically powerful sensory stimuli and abundant in their ecological niche. Sedentary flies, due to the fact that they are already on a favorable niche, don't need *a priori* costly powerful sensory systems as long as they have time to lay eggs and reproduce generations.

## Materials and Methods

### Genetic constructs

Flies were raised in a yeast/agar/propionic acid medium using classic protocols. Double homozygous mutants used in this study were obtained as described in Table S1. *R*, Rover; *S*, Sitter; CG11699*, P-element insertion obtained from the Bloomington Drosophila Stock Center (n. 16374); CG11699**, P-element insertion *EP(X)1556* in *CG11699* from the Berkeley project obtained from Szeged Stock Center (n. 1556, discontinued from November 2009); *CS*, *Canton S*; CG11699* or CG11699**; *for^R^* and CG11699* or CG11699**; *for^S^* correspond to the double homozygotes bearing the *CG11699* mutations in a Rover or Sitter background, respectively. CG11140* has a P-element insertion bearing a *lacZ* in the *Aldh-III* gene and is called Aldh-III* (Bloomington Drosophila Stock Center, n. 11342). This strain, balanced with CyO, is hemizygous. These flies were crossed with Rover flies and the first generation heterozygous flies without balancer were used in the behavior and biochemistry studies. The mutant Rover and Sitter strains were obtained from the laboratory of M. Sokolowski (Toronto University, Canada). The *dnc* and *rut* mutants were obtained from the Bloomington Drosophila Stock Center and the *hsp-PKG* strain derives from a collaborative work with the laboratory of M. Sokolowsky [9].

### Behavioral analysis

An arena (30 cm in diameter and 7 mm in depth) was designed as indicated in a previous publication [56]. This system was set up to generate a gradient of odorants in a controlled manner inside a defined space. The plastic structure of the arena contains four holes (2 mm in diameter), one of which was connected to the odorant source. This structure was then placed in a sandwich between two glass plates. Aldehyde compounds (benzaldehyde, propionaldehyde and acetaldehyde: 100 µl *plus* 1 ml water) were loaded into a 40 ml glass syringe and were used to generate the odors. For the cocktail of the three compounds 33 µl of each were used. The air in the syringe enriched with odors was connected to the arena by a capillary and was injected using an automatic syringe pusher at a speed of 5 ml/minute to create a gradient in the arena before reaching a uniform concentration. Flies were starved for two hr and then placed in the arena 15 min before the start of the experiments, which modalities are indicated in the Figure Legends.

### Plasmids and PCR analysis of transcripts

The *CG11699* cDNA clone (RE61805) of 450 bp was obtained from the Drosophila Genomics Resource Center (Indiana University, Bloomington, IN) and amplified with primers harboring restriction sites at their 5′ ends (5′-AGACACTAGTATGAGCGAGGCCGGCACC-3′ carrying *SpeI* and 5′-ACTAGCGGCCGCCTTCCTTGTTCCAGGC-3′ carrying *NotI*) using *Isis* Taq DNA polymerase (Qbiogene). The PCR products were phosphorylated with the T4 polynucleotide kinase (New England Biolabs) and subcloned into the *SmaI* site of pUC19 (MBI Fermentas). The *Spe*I-*Not I* fragment of this recombinant vector was subsequently subcloned into the copper-inducible pMT/V5-His vector (Invitrogen, Carlsbad, CA) to generate CG11699-pMT/V5-His. For the transcript analysis of *Aldh-III* and of *CG11699*, mRNA was extracted from 50 adult flies and cDNA was synthesized according to the manufacturer's reagents and instructions (Invitrogen). Fragments of the *Aldh-III* cDNA were then amplified with the primers listed in figure S4. Primers 5′-CGCACGCTGGCCACCGCC-3′ (forward) and 5′-TTCCTTGTTCCAGGCTGCC-3′ (reverse) were used in PCR analysis for the detection of *CG11699* in a subclone of S2 cells (see figure S4).

### Cell Culture and transfection studies

Schneider 2 (S2) cells, Schneider medium, fetal bovine serum (FBS), pCoHygro vector and hygromycin-B were purchased from Invitrogen. S2 cells were maintained in Schneider medium supplemented with heat-inactivated 10% (v/v) FBS at 27°C [57]. A stable cell line expressing *CG11699* was generated by transfection of S2 cells with a plasmid bearing CG11699-pMT/V5-His and pCoHygro containing the *E. coli* hygromycin-B phosphotransferase gene under the control of a *Drosophila* Copia promoter (produces selection of resistance to hygromycin-B in S2 cells). The transfected cells were selected with hygromycin-B (300 *µ*g/ml) for four weeks. The stable cells were then treated with 0.5 mM CuSO_4_ for 24 hr to induce *CG11699* expression prior to use. All procedures were performed in accordance with the instruction manual “Drosophila expression system for the stable expression and purification of heterologous proteins in Schneider 2 cells” (Invitrogen, version H, February 28, 2003, 18–19).

### Cell extract analysis

Induced cells were pelleted for 10 min at 1000 xg and re-suspended in PBS. Cells were centrifuged again for 10 min at 13000 xg (at 4°C) and lysed in 50 mM Tris pH 7.8, 150 mM NaCl, 1% Nonidet P-40 for enzymatic dosage. S2 *Drosophila* transfected, induced or control cells were also cultured, then were briefly sonicated (1 min, 50%, *Vibra Cell*, *Bioblock*) to break them. After a brief centrifugation to remove mitochondria, nuclei and organelles (1 min, 1000 rpm, Beckman apparatus), the supernatant enriched in the membrane component was pelleted for 10 min at 13000 xg at 4°C and resuspended in PBS to assay for enzymatic activity. Samples were quantified first for protein content and then immediately used in the assays. For Western blot analysis, protein extracts were separated by 17% SDS-PAGE and the transfer was performed using a semi-dry electroblotter. Detection of recombinant fusion *CG11699* protein was carried out using an anti-V5-HRP antibody (Invitrogen) and the signal was visualized by chemiluminescence.

### Biochemical analyses

Aldehyde dehydrogenase activity was determined using benzaldehyde as the substrate and NAD^+^, NADP^+^ as coenzymes. Aldh enzymatic activity was measured at 25°C in 1 ml of 50 mM sodium phosphate (pH 8) containing 1 mM NAD^+^, 1 mM NADP^+^, 50 µM benzaldehyde, and 0.1 ml of cell extract (100 µg of soluble protein). The production levels of NADH and/or NADPH were determined by measuring the absorbance intensity at 340 nm every five minutes for 45 min using a spectrophotometer equipped with a software module [58], [59], [60], [61]. Crude microsomal preparations (membrane fraction) were used for enzymatic determinations. There are seven *Aldh-III* forms with hydrophobic C-terminal extensions (only one variant lacks this motif) such that this enzyme is mostly bound to the membrane and is particularly active against aliphatic long-chain aldehydes and apolar aldehydes (Aldh-III is sometimes referred to as fatty aldehyde dehydrogenase). For the heat shock experiments, prior to Aldh dosage, flies were placed at 37°C for 20 min twice on day 5 and the enzymatic determination was performed the next day.

## Supporting Information

Figure S1 **Trajectometry analysis of fly exploration stimulated by olfactory cues.** Flies were placed individually in an arena designed so that they could walk but not fly. (A) Four “checkpoint” landmarks were used to count fly passages (black triangle, blue circle and two white oblong shapes). An odorant source such as benzaldehyde (see Materials and Methods) is injected using a push syringe in correspondance of the black triangle at a rate of 5 ml/min (arrow) and the exploration characteristics of the flies are monitored by a camera connected to a software (B) or counted manually (C). (C) The cumulative frequency of passages for ten *Canton S* five day old males (gray) and females (blue), at the four check points, is shown. These experiments were conducted for five minutes during which time the flies are walking and numbers represent the accumulation of ten flies (the periods of time during which the flies are immobile, asleep and/or grooming were not counted). The overall duration of the experiment did not exceed 30 minutes, beyond which time the odorant concentration in the arena becomes equal to the air in the syringe. The scale of the graph represents three values: 30, 60 and 90 passages, and is the same for all the holes of the arena. Shown at the bottom are different alternative transcripts produced by the *for* and *Aldh-III* genes, the two genes analyzed in this olfactory behavioral study.
(TIF)

Figure S2 **Benzaldehyde-induced response of double homozygous mutants bearing another** ***CG11699*** **allele in a Rover or Sitter genetic background.** These representations correspond to a series of experiments carried out in parallel with those represented in figure 2. Another *CG11699* mutant [*(EP)EP* insertion, Berkley Genome project] was tested according to the same protocol except that only five flies were tested. Purple bars represent females and blue bars males. R, Rover; S, Sitter. Bars represent the accumulation of five flies tested individually. The scale of Y-axis corresponds to 15, 30 and 45 passages. Statistical analysis was carried out with a Paired *t*-test for comparison of the groups with the Rover male group (n = 5) as described below in the figure.
(DOC)

Figure S3 **Strain response to grape juice odors.** The average of the timing during which five Rover females performed 25 passages in the triangle was a time reference for the other tested strains. The Y-axis represents the ratio of the time for 25 passages obtained with the other strains compared with the reference. Numbers represent the mean of five trials +/−S.E. A, Rover; B, Sitter; C, CG11699*; D, Aldh-III*; E, CG11699** [(EP)EP insertion]; F, *CS*; G, CG11699*; *for^S^*; H, CG11699*; *for^R^*; I, CG11699**; *for^S^*; J, CG11699**; *for^R^*. The protocol used is the same as that in the other figures and is described in Materials and Methods.
(DOC)

Figure S4 **Primers used in this study and analysis of kinase inhibitors in biochemistry experiments.** S2 cells were stably transfected with an expression vector bearing the *CG11699* coding sequence. A stable cell line expressing *CG11699* was generated by transfection with a plasmid containing CG11699-pMT/V5-His and pCoHygro (the *E. coli* hygromycin-B phosphotransferase gene under the control of a *Drosophila* Copia promoter). Stable cells were treated with 0.5 mM CuSO_4_ for 24 hr to induce *CG11699* expression before use. Transfected cells were broken by sonication and then briefly centrifuged to separate the membrane fraction (pellet) from the soluble component. Membranes (200 µg) were incubated or not with PKA subunit (10 units), ATP (50 µM) and Mg^++^ (1 mM) for 30 min at 30°C in PBS buffer prior to Aldh activity determination (see Materials and Methods). Inhibitors [PKA Inhibitor (6–22), amide, Sigma, 1 µM or Calphostin C, Sigma, 50 nM (specific for PKC at this concentration)] were also co-applied with PKA. The curves represent the average of three determinations.
(DOC)

Figure S5 **Model for Aldh-III activation.** This scheme summarizes the proposed molecular interactions of CG11699 (15 kDa protein with two trans-membrane domains and a short cytosolic sequence bearing putative phosphorylation sites for PKG, PKA and casein kinase) with *Aldh-III* and *for* proteins. The expression of *Aldh-III* is inducible in mammals through xeno-sensors (transcription factors directly activated by xenobiotics). The phosphorylation of CG11699 might regulate the activity of Aldh-III referred by authors as a membrane-cleaning enzyme. Briefly the isoforms of PKG (coded by the gene *for*) expressed in the Rover phenotype might have as substrate the gene product *CG11699*. When phosphorylated, this might bind to some distinct *Aldh-III* protein isoforms and consequently increase its enzymatic activity. CG11699 might also act as an anchor to target Aldh-III activity at specific sites of the cell. We notice that the promoter of the *Aldh-III* gene is under the control of a family of transcription factors, called xenosensors, like the PXR family, which are activated directly by xenobiotic molecules like pesticides or chemical compounds like pharmaceutical drugs. Thus, *Aldh-III* is likely regulated at two levels: one transcriptional, through chemical compounds acting by promoting transcription factor dimerisation, and the other by formation of a complex through phosphorylation, leading to the modulation and targeting of the enzyme.
(DOC)

Table S1 **Crosses used to generate the homozygous** CG11699* **or** CG11699** **;** ***for^R^*** **and** CG11699* **or** CG11699** **;** ***for^S^*** **double mutant flies.** Balancers used: FM6, CyO and Sco.
(DOC)

Table S2 **Statistical analysis of the behavioral studies.** Paired *t*-test statistical analysis of the data presented in figures 2, 3 and 4.
(DOC)
