# Supplementary figures and images for: Trade-Off between Toxicity and Signal Detection Orchestrated by Frequency- and Density-Dependent Genes
Source: PLoS One. 2011 May 19;6(5):e19805. doi: 10.1371/journal.pone.0019805 (PMC3098255; doi:10.1371/journal.pone.0019805)

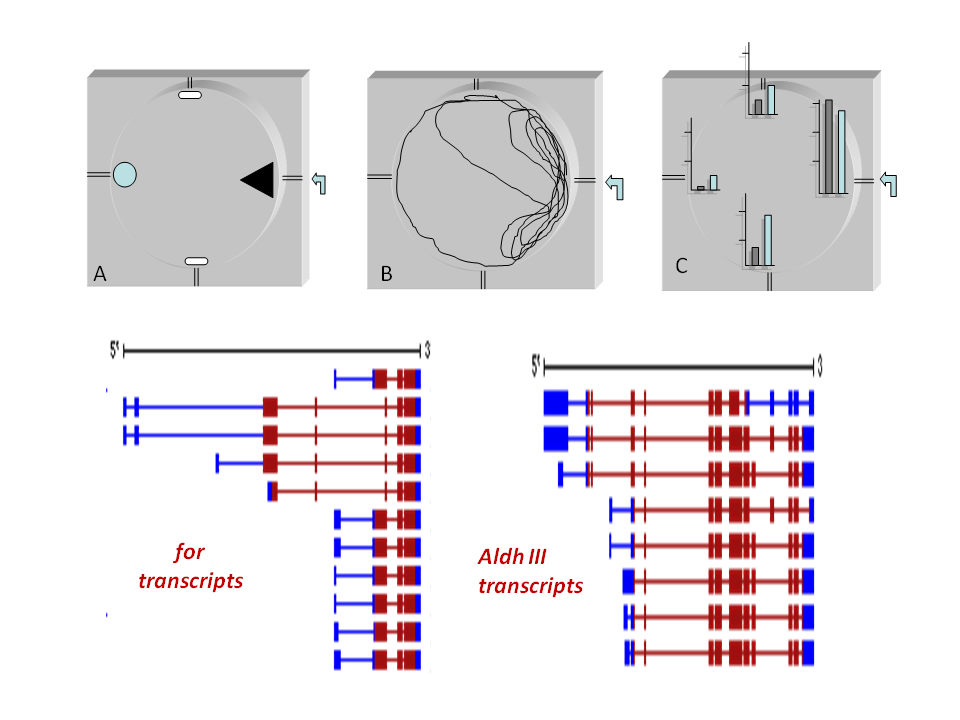

Supplement: Figure S1 — Trajectometry analysis of fly exploration stimulated by olfactory cues. Flies were placed individually in an arena designed so that they could walk but not fly. (A) Four “checkpoint” landmarks were used to count fly passages (black triangle, blue circle and two white oblong shapes). An odorant source such as benzaldehyde (see Materials and Methods) is injected using a push syringe in correspondance of the black triangle at a rate of 5 ml/min (arrow) and the exploration characteristics of the flies are monitored by a camera connected to a software (B) or counted manually (C). (C) The cumulative frequency of passages for ten Canton S five day old males (gray) and females (blue), at the four check points, is shown. These experiments were conducted for five minutes during which time the flies are walking and numbers represent the accumulation of ten flies (the periods of time during which the flies are immobile, asleep and/or grooming were not counted). The overall duration of the experiment did not exceed 30 minutes, beyond which time the odorant concentration in the arena becomes equal to the air in the syringe. The scale of the graph represents three values: 30, 60 and 90 passages, and is the same for all the holes of the arena. Shown at the bottom are different alternative transcripts produced by the for and Aldh-III genes, the two genes analyzed in this olfactory behavioral study. (TIF) [file pone.0019805.s001.tif]
