# Supplementary material for: Trade-Off between Toxicity and Signal Detection Orchestrated by Frequency- and Density-Dependent Genes
Source: PLoS One. 2011 May 19;6(5):e19805. doi: 10.1371/journal.pone.0019805 (PMC3098255; doi:10.1371/journal.pone.0019805)
Supplement: Figure S2 — Benzaldehyde-induced response of double homozygous mutants bearing another CG11699 allele in a Rover or Sitter genetic background. These representations correspond to a series of experiments carried out in parallel with those represented in figure 2. Another CG11699 mutant [(EP)EP insertion, Berkley Genome project] was tested according to the same protocol except that only five flies were tested. Purple bars represent females and blue bars males. R, Rover; S, Sitter. Bars represent the accumulation of five flies tested individually. The scale of Y-axis corresponds to 15, 30 and 45 passages. Statistical analysis was carried out with a Paired t-test for comparison of the groups with the Rover male group (n = 5) as described below in the figure. (DOC) [file pone.0019805.s002.doc]

On the oblong:

**Rover** male *versus* **CG**; S** male; T value: 1.8; P value: 0.14; degree of freedom: 4

**Rover** male *versus* **CG**; S** female; T value: 2.23; P value: 0.08; degree of freedom: 4

**Rover** male *versus* **CG**; R** male; T value: 1.61; P value: 0.18; degree of freedom: 4

**Rover** male *versus* **CG**; R** female; T value: 1.24; P value: 0.28; degree of freedom: 4

**Rover** male *versus* **CG**** male; T value: 4,35; P value: 0.012; degree of freedom: 4

**Rover** male versus **CG**** female: T value: 4.8; P value: 0.009; degree of freedom: 4

On the triangle:

**Rover** *versus* **CG****, male; T. Value: 6.16; P value: 0.0016; degree of freedom: 5

**Rover** male *versus* **CG**** female; T. Value 7; P value: 0.000916; degree of freedom: 5

**Rover** male versus **CG**; R** male; T. Value 2.71; P value: 0.0418; degree of freedom: 5

**Rover** male *versus* **CG**; R** female; T value 7.36; P value 0.000726; degree of freedom: 5

**Rover** male *versus* **CG**; S** male; T value: 2.388; P value: 0.0625; degree of freedom: 5

**Rover** male *versus* **CG**; S** female; T value: 3.529; P value: 0.0167; degree of freedom: 5
