# Supplementary material for: Trade-Off between Toxicity and Signal Detection Orchestrated by Frequency- and Density-Dependent Genes
Source: PLoS One. 2011 May 19;6(5):e19805. doi: 10.1371/journal.pone.0019805 (PMC3098255; doi:10.1371/journal.pone.0019805)
Supplement: Figure S4 — Primers used in this study and analysis of kinase inhibitors in biochemistry experiments. S2 cells were stably transfected with an expression vector bearing the CG11699 coding sequence. A stable cell line expressing CG11699 was generated by transfection with a plasmid containing CG11699-pMT/V5-His and pCoHygro (the E. coli hygromycin-B phosphotransferase gene under the control of a Drosophila Copia promoter). Stable cells were treated with 0.5 mM CuSO4 for 24 hr to induce CG11699 expression before use. Transfected cells were broken by sonication and then briefly centrifuged to separate the membrane fraction (pellet) from the soluble component. Membranes (200 µg) were incubated or not with PKA subunit (10 units), ATP (50 µM) and Mg++ (1 mM) for 30 min at 30°C in PBS buffer prior to Aldh activity determination (see Materials and Methods). Inhibitors [PKA Inhibitor (6–22), amide, Sigma, 1 µM or Calphostin C, Sigma, 50 nM (specific for PKC at this concentration)] were also co-applied with PKA. The curves represent the average of three determinations. (DOC) [file pone.0019805.s004.doc]

**Primers used in this study corresponding to *AldhIII* gene**

Aldh1 (forward)

TTACACTAGTTCCGACAGAATGGCC

Aldh2 (forward)

CATTCTCGAGCGCTTCGACAACAGCTTGC

Aldh3 (forward)

TGACACTAGTACTACGATAATGTTTGACAACG

Aldh4 (forward)

CGTTCTCGAGCGATCAGTTGAGCTTTTCTGTAG

Aldh5 (reverse)

GAGCTGGAATAGGATGTGCC

Aldh6 (reverse)

CTGCCCTTCGGAGGCGTCGG

cDNA molecules of the *Aldh III* gene were synthesized and the arrows indicate the location of the primers on the exons for amplification.

**Primers CG11699**

CDS CG11699

TCAAGAGCGGTCAAGCAATCAGCTGTGTTGTGCGATCCAATTCGGGCATTAGTAGTTACGACAACAATAAATCGCAATTGAGAAGGATGAGCGAGGCCGGCACCAGCGCGGATGCAGTGGCCGCCGAGAAGGAGCGCAAGTTCCGCATCCAGGgtaggtggttctccaaacacatacagacacacgcatatatatcagccagcaactgactgaagtacaactccatttcatcctcctttctagCCGCCGCCTTTCTGGGTTTGGTGGGCGGGGTGTCCGCTCTGTTCGGCTTCTCGCGCACGCTGGCCACCGCCAAGAAGACGGATAGCAAGGTCCTCCAGCAGGCTGGAACGCGGCAAGGCATGATCCTGATGGACGAGGGCGCCACCCTGGCCCTGCGGGCGCTGGGCTGGGGCACACTGTACGCCGTGATGGGCACCGGCGCCTTCTGCTACGGCTTCTGGAAGCTATCCGGAGCCAAGGATgtgagcatgcctccgatttgttcaatgaatttcgctttaaatctttcgtttcgttcgcagTTCCAGGAGTTCCGCCTCAAGATGGGCAATGCACTGCCAAGAATCACCAAGGACGAACCACCAGCCAGCCGCACCGATTTCGAGAGTCTCACGGACCTCATGAAATACCTGGCAG CCTGGAACAAGGAATAAGCAGCTCACAAATATACACACAATTTAAAGTAGACCTAACGCCTGAGTAGACCAGAACATAAAACGAAACCTTTGTTAACTTCAGTTTACATACAGACAATGT TTTCAAGAC

ctggttctccaaa : intron

The underlined sequences correspond to the location of primers used in this study.

CGCACGCTGGCCACCGCC (forward)

CCGTCGGACCTTGTTCCTT (reverse)

**Putative phosphorylation sites in CG11699:**

|  |
| --- |

**NetPhosK 1.0 Server-prediction results for phosphorylation**

Method: NetPhosK without ESS filtering:

Query: Sequence CG11699

Site Kinase Score

---------------------

T-45 PKC 0.90

T-49 PKC 0.58

T-81 DNAPK 0.60

S-99 PKA 0.51

T-119 PKG 0.53

T-128 CKII 0.53

T-128 PKG 0.53

S-132 CKI 0.60

T-134 CKII 0.56

---------------------

Highest Score: 0.90 PKC at position 45

# CG11699 [Drosophila melanogaster]

MSEAGTSADAVAAEKERKFRIQAAAFLGLVGGVSALFGFSRTLATAKKTDSKVLQQAGTRQGMILMDEGA

TLALRALGWGTLYAVMGTGAFCYGFWKLSGAKDFQEFRLKMGNALPRITKDEPPASRTDFESLTDLMKYL

AAWNKE
