# Supplementary material for: Trade-Off between Toxicity and Signal Detection Orchestrated by Frequency- and Density-Dependent Genes
Source: PLoS One. 2011 May 19;6(5):e19805. doi: 10.1371/journal.pone.0019805 (PMC3098255; doi:10.1371/journal.pone.0019805)
Supplement: Table S1 — Crosses used to generate the homozygous CG11699* or CG11699** ; forR and CG11699* or CG11699** ; forS double mutant flies. Balancers used: FM6, CyO and Sco. (DOC) [file pone.0019805.s006.doc]

1. ***1: mutation on the X (CG11699* or CG11699**)**

# ♀  *1 /*1 ; +/+ × ♂  FM6/Y ; CyO/Sco

# F1 :

# ♀ *1 /FM6; +/CyO and *1 /FM6 ; +/Sco

# ♂ *1 /Y ; +/CyO and *1 /Y; +/Sco

# F2:

# ♀ *1 /*1 ; CyO/Sco and ♂ *1 /Y ; CyO/Sco

1. ***2: mutation on 2L (*for^R^* or *for^S^*)**

♀  FM6 /FM6;  CyO/Sco X ♂  +/Y ; *2/ *2

F1 :

♀ FM6/+ ; *2/ CyO and FM6/+ ; *2/ Sco

♂ FM6/Y ; *2/ CyO and FM6/Y ; *2/ Sco

F2:

**FM6/Y ; *2/*2**

Final cross

♀  *1 /*1 ; CyO/Sco × ♂  FM6/Y; *2/*2

# F1: *1/FM6;  *2/ CyO and *1/Y; *2/ Sco

F2:

***1 /*1 ; *2/*2** and ***1 /Y ; *2/*2**
