# Supplementary material for: Trade-Off between Toxicity and Signal Detection Orchestrated by Frequency- and Density-Dependent Genes
Source: PLoS One. 2011 May 19;6(5):e19805. doi: 10.1371/journal.pone.0019805 (PMC3098255; doi:10.1371/journal.pone.0019805)
Supplement: Table S2 — Statistical analysis of the behavioral studies. Paired t-test statistical analysis of the data presented in figures 2, 3 and 4. (DOC) [file pone.0019805.s007.doc]

**Statistical analysis**

**Figure 2 using Paired t test:**

On the oblong:

**Rover** *versus* **Sitter** male. T value: 0.77; P value: 0.46; degree of freedom: 9

**Rover** *versus* **Sitter** female. T value: 5.579; P value: 0.00034; degree of freedom: 9

**Rover** female *versus* male. T value: 1.59; P value: 0.1448; degree of freedom: 9

**Sitter** female *versus* male. T value: 3.92; P value: 0.0035; degree of freedom: 9

**Rover** *versus* **CG*** male. T value: 3.62; P value: 0.00556; degree of freedom: 9

**Rover** *versus* **Aldh*** male. T value: 3.92; P value: 0.00349; degree freedom: 9

**Rover** *versus* **CG*; R**male. T value: 1.762; P value: 0.1288; degree of freedom: 9

**Rover** *versus* **CG*; S** male. T value: 4.738; P value: 0.001; degree of freedom: 9

**Rover** *versus* **CG*** female. T value: 1.69; P value: 0.247; degree of freedom: 9

**Rover** *versus* **Aldh*** female. T value: 4.725; P value: 0.001; degree of freedom: 9

**Rover** *versus* **CG*; R** female. T value: 2.58; P value: 0.0296; degree of freedom: 9

**Rover** *versus* **CG*; S** female. T value: 2.68; P value:0.025; degree of freedom: 9

On the triangle:

**Rover** *versus* **Sitter** male. T value: 1.123; P value: 0.29; degree of freedom: 9

**Rover** *versus* **Sitter** female. T value: 0.49; P value: 0.63; degree of freedom: 9

**Rover** female *versus* male. T value: 1.49; P value: 0.18; degree of freedom: 9

**Sitter** female *versus* male. T value: 0.38; P value: 0.7; degree of freedom: 9

**Rover** *versus* **CG*** male. T value: 1.84; P value: 0.098; degree of freedom: 9

**Rover** *versus* **Aldh*** male. T value: 6.14; P value: 0.00017; degree freedom: 9

**Rover** *versus* **CG*; R** male. T value: 2.32; P value: 0.045; degree of freedom: 9

**Rover** *versus* **CG*; S** male. T value: 4.47; P value: 0.00152; degree of freedom: 9

**Rover** *versus* **CG*** female. T value: 6.51; P value: 0.0001; degree of freedom: 9

**Rover** *versus* **Aldh-III*** female: T value: 6.21; P value: 0.00015; degree of freedom: 9

**Rover** *versus* **CG*; R** female. T value: 4.58; P value: 0.0013; degree of freedom: 9

**Rover** *versus* **CG*; S** female. T value: 7.67; P value:0.00003; degree of freedom: 9

**Figure 3 using Paired t test:**

**Rover** male *versus* female. T value: 2.8; P value: 0.00228; degree of freedom: 8

**Sitter** male *versus* female. T value: 3; P value: 0.00168; degree of freedom: 8

**CS** male *versus* female. T value: 4.54; P value: 0.00189; degree of freedom: 8

**Rover** *versus* **Aldh-III*** female. T value: 5.17; P value: 0.000846; degree of freedom: 8

**Rover** *versus* **CG*** female. T value: 4.81; P value: 0.00132; degree of freedom: 8

**Rover** *versus* **CG*; S** female T value: 2.889; P value: 0.00963; degree of freedom: 8

**Rover** *versus* **CG***; **R** female. T value: 3.38; P value: 0.00963; degree of freedom: 8

**Rover** *versus* **Aldh-III*** male. T value: 0.225; P value: 0.827; degree of freedom: 8

**Rover** *versus* **CG*** male. T value: 0.9; P value: 0.39; degree of freedom: 8

**Rover** *versus* **CG*; S** male. T value: 0.064; P value: 0.95; degree of freedom: 8

**Rover** *versus* **CG*; R** male. T value: 0.034; P value: 0.97; degree of freedom: 8

Statistics of the Rover, Sitter and CS female strains *versus* the mutants *Aldh*,* *CG11699** and the double mutants *CG11699*; R* and *CG11699*; S* female or male are not reported.

**Figure 4 using Paired t test:**

**Rover** female *versus* male. T value: 2.04; P value: 0.1; degree of freedom: 4

**Rover** female *versus* **Sitter** female. T value: 0.16; P value: 0.8; degree of freedom: 4

**Rove**r female *versus* **CS** female. T value 1.136; P value: 0.319; degree of freedom: 4

**Rover** female *versus* **Aldh-III*** female. T value:9.9 P value 0.000579; degree of freedom: 4

**Rover** female *versus* **CG*** female. T value: 9; P value 0.00078; degree of freedom: 4

**Rover** female *versus* **CG*; R** or **S**. T value: 11; P value:0.00036; degree of freedom: 4
