# Supplementary material for: Trade-Off between Toxicity and Signal Detection Orchestrated by Frequency- and Density-Dependent Genes
Source: PLoS One. 2011 May 19;6(5):e19805. doi: 10.1371/journal.pone.0019805 (PMC3098255; doi:10.1371/journal.pone.0019805)

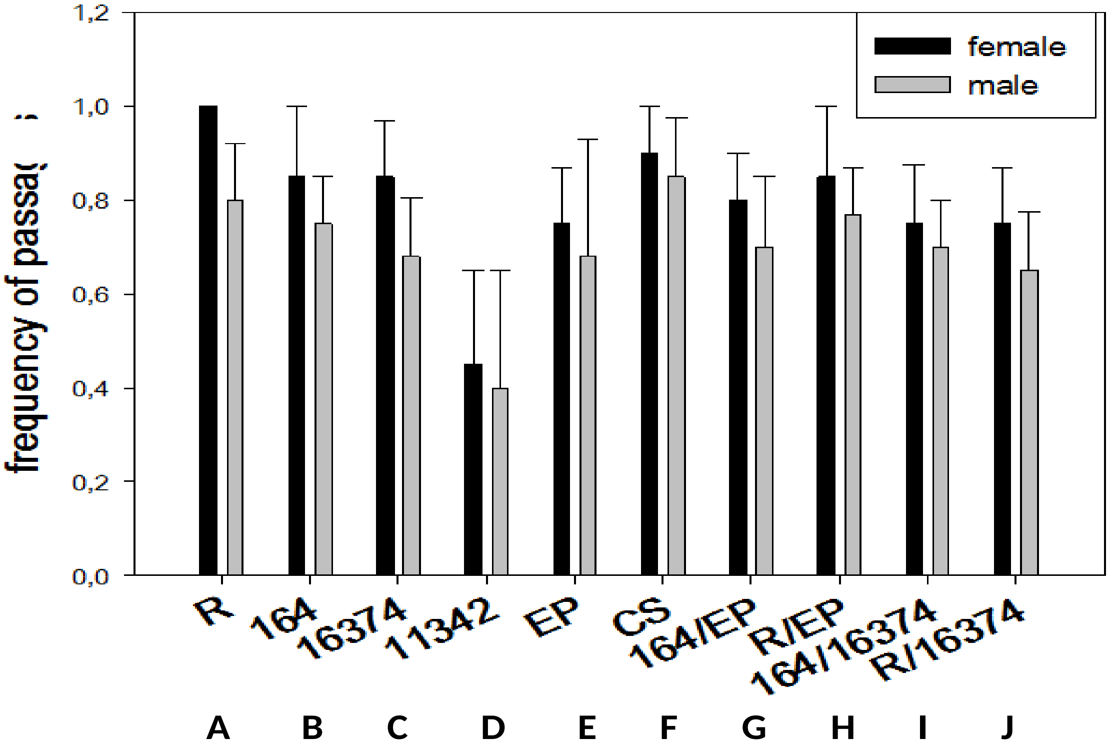
1,2
1,0
0,8
0,6
0,4
0,2
0,0
female
male
R
164
16374
11342
EP
CS
164/EP
R/EP
164/16374
R/16374
A
B
C
D
E
F
G
H
I
J

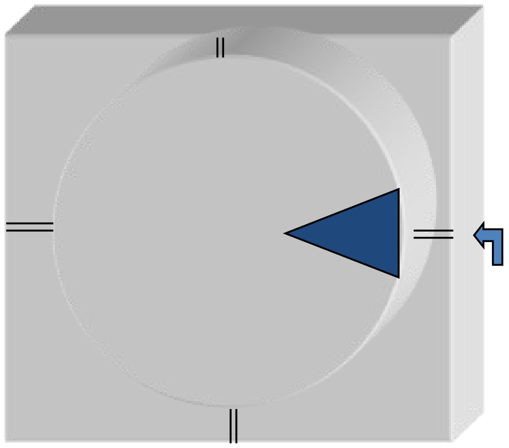

Supplement: Supplementary file 6 [file pone.0019805.s003.doc]
