# Supplementary material for: Trade-Off between Toxicity and Signal Detection Orchestrated by Frequency- and Density-Dependent Genes
Source: PLoS One. 2011 May 19;6(5):e19805. doi: 10.1371/journal.pone.0019805 (PMC3098255; doi:10.1371/journal.pone.0019805)
Supplement: Supplementary file 7 [file pone.0019805.s005.doc]

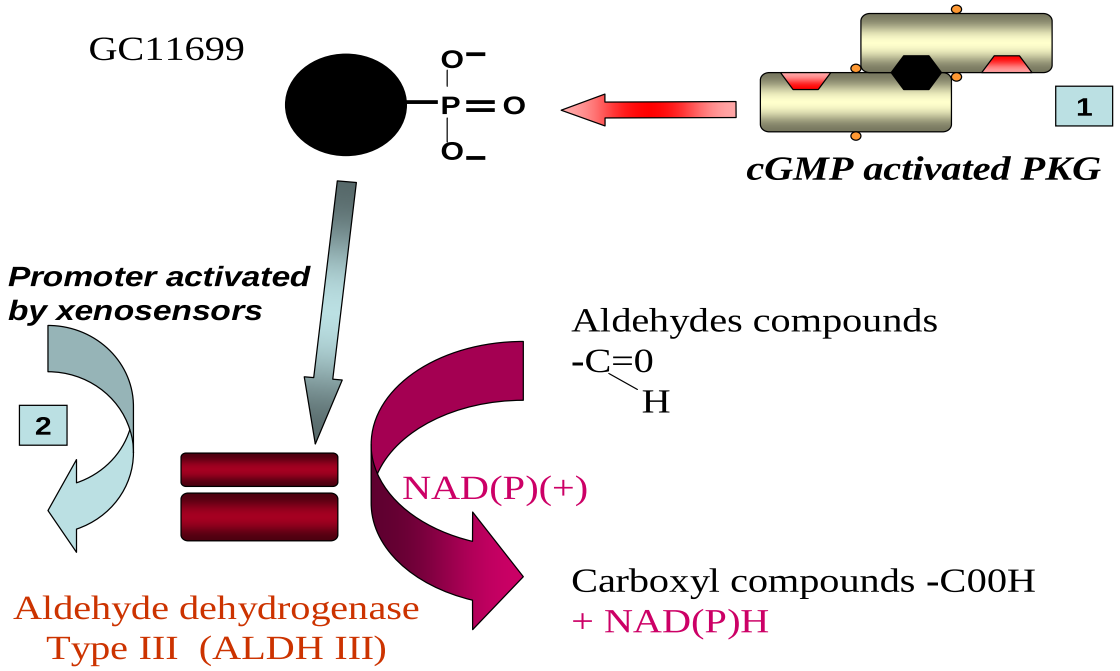

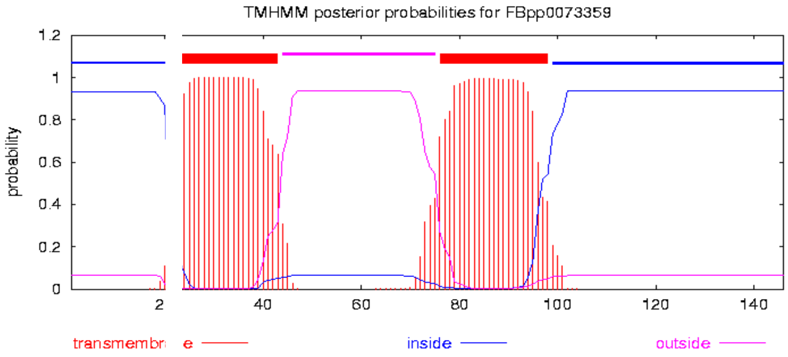

Serine (position,matching motif)
S132 CK1[S/T-X-X-X-S

Threonine (position, matching motif)
T119 PKA [R-X-S/T]; CK2 [S/T-X-X-E]
T128 CK2[S/T-X-X-E]
